# Short and long-term outcomes of patients with COVID-19-associated acute respiratory distress syndrome and difficult veno-venous-ECMO weaning

**DOI:** 10.1186/s13054-021-03758-4

**Published:** 2021-09-16

**Authors:** Paul Masi, Samuel Tuffet, Laurent Boyer, Thierry Folliguet, Armand Mekontso Dessap, Nicolas de Prost

**Affiliations:** 1 Service de Médecine Intensive Réanimation, DMU MEDECINE, AP-HP, Hôpitaux Universitaires Henri-Mondor, 51, Avenue du Maréchal de Lattre de Tassigny, 94010 Créteil, France; 2 Université Paris Est Créteil, Groupe de Recherche Clinique CARMAS, 94010 Créteil, France; 3 Département de Physiologie-Explorations Fonctionnelles, AP-HP, Hôpitaux Universitaures Henri Mondor, DHU-ATVB, Créteil, France; 4 Département de Pneumologie et Pathologie Professionnelle, Centre Hospitalier Intercommunal, DHU-ATVB, Créteil, France; 5 Service de Chirurgie Cardiaque, DMU CARE, Assistance Publique-Hôpitaux de Paris (AP-HP), Hôpitaux Universitaires Henri Mondor, 94010 Créteil, France; 6 Faculté de Santé, Université Paris Est Créteil, 94010 Créteil, France

To the editor,

The 2019 coronavirus pandemic induced a massive influx of patients with acute respiratory distress syndrome [1], a part of them requiring veno-venous (VV)-extra-corporeal membrane oxygenation (ECMO) support [2]. A consensus of experts has recently published recommendations on VV-ECMO weaning [3, 4], derived from the EOLIA trial [5]. VV-ECMO weaning should be tested when native lung function has sufficiently recovered, allowing for adequate oxygenation and protective mechanical ventilation [e.g., ventilator FiO_2_ ≤ 60%, tidal volume ≥ 6 mL/kg of predicted body weight (PBW), respiratory rate ≤ 28/min, plateau pressure (*P*_plat_) ≤ 28 cmH_2_O)]. Success criteria of a weaning test (with the membrane ventilation decreased to 0 L/min) for safe decannulation from ECMO are typically as follows: PaO_2_ ≥ 60 mmHg and PaCO_2_ ≤ 50 mmHg or pH ≥ 7.36 with ventilator FiO_2_ ≤ 60% and protective mechanical ventilation. However, some patients may undergo ECMO decannulation without meeting readiness to wean criteria and/or succeeding the weaning test.

The aim of this monocentre retrospective cohort study was to report the outcome of patients who underwent a conventional ECMO weaning (withdrawal after readiness to wean and successful weaning test as per EOLIA criteria) [5] to that of patients who underwent an unconventional facilitative weaning (because of a serious complication of VV-ECMO or lack of respiratory system mechanics improvement despite prolonged support (i.e., ≥ 10 days) in patients who have recovered a satisfactory native lung oxygenation, which justifies withdrawal despite no readiness to wean and/or unsuccessful weaning test). No other treatment was discontinued after ECMO weaning. Fifty-one COVID-19 patients admitted between March 2020 and June 2021 in our French tertiary center who required VV-ECMO support were included in the study. Seventeen patients (33%) died on VV-ECMO, whereas 34 (67%) were weaned off VV-ECMO, including 30 who were discharged alive from our ICU (three patients died and one is still in our ICU). Eighteen patients presented the criteria for facilitative weaning while 16 underwent conventional weaning. VV-ECMO weaning was justified in the facilitative group by one or more of the following: major bleeding (*n* = 5), infection (*n* = 2), severe hemolysis (*n* = 2), no respiratory function improvement despite prolonged duration of VV-ECMO support (*n* = 12, median [interquartile range 25–75] duration: 24 days [13–43]). Patients of the facilitative weaning group had more complications before VV-ECMO weaning, more often required prone position after VV-ECMO withdrawal, and had longer mechanical ventilation support and ICU length of stay than their counterparts (Table 1). Only two patients with facilitative weaning and one patient with conventional weaning died in the ICU. Strikingly, respiratory system mechanics, gas exchanges and CT-scan were more impaired at the time VV-ECMO was weaned off with facilitative *versus* conventional strategy (Table 1), consistent with a lung fibrosing process in the former group. Notably, the high plateau and driving pressure levels measured in this group were observed while ventilating patients with low tidal volumes as 75% of these were receiving less than 6 mL/kg PBW. Interestingly, no differences were observed regarding echocardiography, pulmonary function tests and chest CT-scan patterns of lung fibrosis in a subgroup of patients followed-up until 3–6 months of hospital discharge, except for more traction bronchiectasis in patients who underwent facilitative weaning (Table 2).

**Table 1:** Patients’ characteristics and outcomes in the intensive care unit of patients with conventional or facilitative ECMO weaning

Parameters	Facilitative weaning (*n* = 18)	Conventional weaning (*n* = 16)	*P* value
Age, years	53 (45–57)	50 (44–58)	0.92
Male gender (%)	11 (61)	11 (69)	0.70
SAPS 2 score	35 (27–54)	35 (29–50)	0.98
BMI, kg/m^2^	29.1 (26.1–31.9)	34.5 (26.10–35.8)	0.40
*Between ICU admission and ECMO weaning*			
History of previous lung disease	0 (0)	1 (6)	0.47
Chest CT-scan upon ICU admission			
Pulmonary embolism	1 (6)	2 (13)	0.59
Lung parenchyma affected, %	68 (50–90)^a^	75 (50–75)^b^	0.50
Corticosteroids during ICU stay			
Dexamethasone	11 (61)	9 (56)	> 0.99
Hydrocortisone/Fludrocortisone	8 (44)	4 (33)	0.30
Methylprednisolone pulse therapy	2 (11)	1 (6)	> 0.99
Renal replacement therapy	7 (39)	5 (31)	0.73
Ventilator-associated pneumonia	17 (94)	10 (63)	0.030
Major bleeding^c^	13 (72)	4 (25)	0.015
ECMO support duration, days	24 (16–43)	10 (7–14)	< 0.001
*At time of ECMO weaning trial*			
Ventilator settings during ECMO weaning			
Tidal volume, mL	345 (308–396)	400 (320–442)	0.10
Tidal Volume, mL/kg PBW	5.6 (4.8–5.9)	5.8 (5.5–6.1)	0.20
Respiratory rate, breaths/min	34 (30–38)	29 (26–32)	0.002
Plateau pressure, cmH_2_O	31 (29–34)	25 (22–26)	< 0.001
Driving pressure, cmH_2_O	24 (22–27)	13 (12–16)	< 0.001
RS compliance, mL/cmH_2_O	14 (12–17)	27 (22–35)	< 0.001
PEEP, cmH_2_O	5 (5–8)	10 (7–12)	0.003
Arterial blood gases during weaning			
pH	7.35 (7.27–7.38)	7.42 (7.36–7.44)	0.008
PaCO_2_, mmHg	47 (42–55)	41 (37–44)	0.001
PaO_2_, mmHg	82 (71–96)	84 (76–104)	0.37
Arterial lactate levels, mmol/L	0.9 (0.6–1.2)	1.1 (0.9–1.4)	0.10
HCO3^−^, mmol/L	27 (24–29)	27 (23–29)	0.90
PaO_2_/FiO_2_ ratio, mmHg	166 (145–202)	200 (156–254)	0.25
FiO_2_	50 (40–60)	50 (40–50)	0.29
BAL fluid cytological analysis^d^			
Total cell counts; 10^3^/mL	474 (240–772)	500 (259–873)	0.90
Macrophages, %	27 (12–48)	75 (18–89)	0.15
Neutrophils, %	50 (27–71)	18 (5–50)	0.07
Lymphocytes, %	9 (2–17)	4 (3–35)	0.67
Eosinophils, %	1 (0–3)	0 (0–2)	0.24
Chest CT-scan at time of weaning^e^			
Reticular pattern	3 (21)	1 (8)	0.60
Ground glass opacity	11 (78)	12 (100)	0.13
Alveolar condensation	12 (86)	9 (75)	0.53
Traction bronchiectasis	12 (86)	5 (12)	0.038
Tracheal distorsion	1 (7)	0 (0)	> 0.99
Scissural distortion	4 (29)	2 (16)	0.59
*After ECMO withdrawal*			
MV with non-protective settings^f^, days	6 (4–10)	1 (0–2)	< 0.0001
Rescue therapy after weaning			
Prone positioning	9 (50)	1 (6)	0.008
Inhaled nitric oxide	4 (22)	1 (6)	0.34
Methylprednisolone pulse therapy	1 (5)	0 (0)	–
RS mechanics on the day of MV weaning			
Pressure support level, cm H_2_O	11 (8–14)	10 (8–13)	0.60
Tidal volume, mL	520 (411–609)	471 (397–622)	0.75
Tidal volume, mL/kg PBW	7.2 (6.3–8.4)	7.0 (5.9–8.7)	0.98
Compliance^g^, mL/cmH_2_O	44.7 (35.2–62.4)	48.9 (34.1–77.8)	0.78
Total MV duration, days	55 (38–86)^h^	21 (14–31)	0.0002
MV duration after ECMO weaning, days	26 (16–36)^h^	5 (3–12)	< 0.0001
ICU length of stay, days	55 (40–91)^h^	27 (19–32)	< 0.0001
In-ICU mortality	2 (13)^h^	1 (6)	0.60

Continuous variables are expressed as median (interquartile range) and were compared with the Mann–Whitney test; Categorical variables are expressed as n (%) and were compared with *χ*^2^ or Fischer tests, as appropriate

^a^Available for 14 patients

^b^Available for 13 patients

^c^Major bleeding defined by Bleeding Academic Research Consortium (BARC) consensus classification type 3 or more; *SAPS 2* Simplified Acute Physiology Score 2, *BMI* body mass index, *ECMO* extracorporeal membrane oxygenation, *ICU* intensive care unit, *MV* mechanical ventilation, *PBW* predicted body weight, *RS* respiratory system, *PEEP* positive end-expiratory pressure

^d^Available for 12 patients in the facilitative weaning group and 5 patients in the conventional weaning group

^e^Available for 14 patients in the facilitative weaning group and 12 patients in the conventional weaning group

^f^Defined by the number of days with a plateau pressure ≥ 30 cm H_2_O and/or a driving pressure > 15 cm H_2_O

^g^Computed as tidal volume (mL)/pressure support level (cm H_2_O)

^h^One patient was still in the ICU at the time of this report

**Table 2:** Long-term outcomes (three to six months after hospital discharge) of patients with conventional or facilitative weaning

	Facilitative weaning (*n* = 6)	Conventional weaning (*n* = 7)	*P* value
Pulmonary hypertension^a^	0 (0)	0 (0)	–
Pulmonary function tests			
K_CO_, % predicted	88 (75–100)	104 (88–111)	0.11
DL_CO_, % predicted	57 (44–73)	70 (57–72)	0.29
FVC % predicted	77 (59–85)	82 (52–91)	0.92
TLC, % predicted	75 (65–79)	77 (64–94)	0.70
Chest CT-scan at long-term			
Reticular pattern	1 (12)	1 (14)	> 0.99
Ground glass opacity	5 (71)	4 (50)	0.60
Alveolar condensation	0 (0)	1 (12.5)	> 0.99
Traction bronchiectasis	4 (57)	4 (50)	> 0.99
Tracheal traction	0 (0)	0 (0)	–
Scissural distortion	2 (29)	1 (13)	0.57
6-min walking test			
Walked distance, m	433 (348–503)	506 (480–548)	0.08
% of predicted distance, %	67 (62–74)	90 (78–97)	0.009
Room air saturation	97 (96–98)	98 (96–98)	0.82
Dyspnea (MRC scale)			0.07
0	0 (0)	4 (57)	
1 or 2	6 (100)	3 (43)	

^a^Assessed by transthoracic echocardiography; *KCO* CO transfer coefficient*; D*_*LCO*_ haemoglobin value (Hb) corrected diffusion capacity with CO; *FVC* forced expiratory vital capacity; *TLC* total lung capacity

Despite they did not meet the classical weaning criteria [3, 4], patients with facilitative weaning had a low ICU mortality. At long-term follow-up, they also showed good recovery on pulmonary function tests and chest CT imaging. These data illustrate that VV-ECMO withdrawal criteria could be less restrictive, especially in patients developing life-threatening complications under VV-ECMO support or with reasonable recovery of native lung oxygenation function but no improvement of respiratory system mechanics. Our results need to be confirmed and the best ventilator settings to be applied after ECMO weaning to be further studied.

## Data Availability

The dataset used during the current study is available from the corresponding author upon reasonable request.

## Abbreviations

BMI: Body mass index
CT: Computerized tomography
DL_CO_: Haemoglobin value corrected diffusion capacity with CO
FVC: Forced expiratory vital capacity
ICU: Intensive care unit
KCO: CO transfer coefficient
MV: Mechanical ventilation
PBW: Predicted body weight
PEEP: Positive end-expiratory pressure
*P*_plat_: Plateau pressure
RS: Respiratory system
SAPS: Simplified acute physiology score 2
TLC: Total lung capacity
VV-ECMO: Veno-venous extra-corporeal membrane oxygenation
